# Decreasing the carbon footprint of food through public procurement—A case study from the municipality of Härnösand

**DOI:** 10.3389/fnut.2024.1330892

**Published:** 2024-11-06

**Authors:** Mari Kjellberg, Wilhelm Skoglund, Henrik Haller

**Affiliations:** ^1^Department of Natural Science, Design and Sustainable Development (NDH), Mid Sweden University, Östersund, Sweden; ^2^Department of Economics, Geography, Law, and Tourism (EJT), Mid Sweden University, Östersund, Sweden

**Keywords:** food policy, decarbonization, Sweden, protein shift, eating habits

## Abstract

Eating habits are among the strongest drivers of negative environmental impact. Public procurement has been suggested as an efficient lever to catalyze changes within the food system. This study examines alternative purchase processes that may decrease the carbon footprint of publicly procured food through a case study of a municipality in the Northern part of Sweden. The GHG emissions associated with the current food service in the case study were 2.2 kg CO_2_e per kg food and must be reduced by 40.9% by 2030 to comply with the Paris Agreement; 76% of the emissions derive from food of animal origin (44% from unprocessed red meat). Three alternative diet scenarios, “zero red meat,” “−50% red meat,” and “flexitarian free from red meat,” were explored. Only 6% of the total purchased food kilograms were altered, yet the cutback of meat caused GHG emissions reductions turned out to be as high as 44%. The Swedish Law on Public Procurement, deficient infrastructure, unsustainable food culture, and local politicians' reluctance to change were mentioned as the main obstacles to materializing necessary changes in the food procurement system. The respondents also pointed out essential policy changes at the national and municipal levels.

## 1 Introduction

Although the Paris Agreement is ratified or acceded by 194 states and the EU, the commitments of most of the parties, in terms of decarbonization, are insufficient to achieve the goals of this legally binding international treaty (1). The food system, i.e., all elements and activities related to producing and consuming food, and their effects, is the largest pressure caused by humans on the earth's ecosystems (2) and accounts for up to 34% of the total greenhouse gas (GHG) emissions globally (3). Eating habits have been pointed out as one of the strongest drivers of climate change, loss of biodiversity, loss of drinking water, disruption of the global nitrogen and phosphorus cycles, and deforestation (2, 4–6). Rockström et al. argue that neither the UN sustainable development goals nor the Paris Climate Agreement can be met without transforming the food system (7). To stay within the safe operating space of the planetary boundaries and to achieve the global goals for sustainable development, it is therefore essential that the food system is reviewed and transformed toward sustainability (6, 8, 9). All stages of the food system (growing, harvesting, processing, packaging, transporting, marketing, consumption, distribution, and disposal of food) need a transformation to different degrees (6).

The large GHG emissions of the food sector stress the need for strategies to reduce these by shifting food production systems and dietary patterns. To reach climate targets, a shift from a diet high in animal proteins to plant-based diets, especially in industrialized countries, is increasingly acknowledged as crucial (10–15). As a suggestion for a healthy diet and that can be produced within the planetary boundaries, the Lancet Commission have developed the “planetary health diet.” The suggested diet matches the optimal caloric intake, and the daily intake consists largely of a diversity of plant-based foods and low amounts of animal-source foods. Such a diet contains unsaturated rather than saturated fats, limited amounts of refined grains, highly processed foods, and added sugars and is produced within the biophysical planetary boundaries (16–18). The Commission states that, with such a diet, it is possible to provide healthy food to a population of 10 billion people within the framework of the planetary boundaries by the year 2050. This, however, will require an immediate and comprehensive transformation of the food system (2). This transition entails several dietary changes such as (1) a reduction in the intake of animal protein (meat, eggs, and dairy products), (2) an increased share of regionally produced, in-season food, (3) an increased proportion of organic produce, and (4) that food that has been produced as energy efficiently as possible is prioritized (2, 5, 12, 13, 19).

Public procurement, i.e., all purchases made by public authorities, such as government departments or local authorities, has been suggested as an efficient lever to catalyze the necessary changes within the food system (9, 20–23), and Swedish civil servants have identified public procurement as a policy area that could be used to advance the consumption perspective on sustainability (24). Public procurement is also an important instrument for achieving target 12:7 of the UN Sustainable Development Goals (9). There is an increasing body of research providing a narrative of potentially increased sustainability through public food procurement (PFP), but much less research has been conducted on the outcomes in terms of sustainability from different procurement processes (21, 22, 24). Molin et al. point out that there is a need for studies addressing how to improve the public procurement processes (21), and Morley states that there is not sufficient research on the potential of public procurement to influence the food-producing sector (22). The total value of procurement within the public sector in Sweden is 18.3% of the gross domestic product (GDP) in total (25), and the worth of public procurement of food is 10 billion SEK (≈9 million USD) per year from serving 3 million meals a day. In Härnösand, the municipality explored in this study, the turnover of public procured food is 24 million SEK (≈2.1 million USD), and 1.1 million meals are served per year (schools and retirement homes included, but hospitals excluded).

With the overall ambition to continue building upon previous knowledge and research within food systems and decarbonization, the objectives of this study were primarily to identify the main contributors to GHG emissions within publicly procured food in the municipality of Härnösand and, second, to evaluate different climate mitigation scenarios (“zero red meat,” “−50% red meat,” and “flexitarian free from red meat”) aiming to decreasing the carbon footprint through strategic replacements of animal protein by less carbon-intense alternatives. Third, obstacles and enablers for materializing such a dietary shift in public gastronomy were assessed.

## 2 Methods

### 2.1 Geographical context

This carbon footprint was calculated for the publicly procured food in the municipality of Härnösand (≈25,000 habitants) in the Northern part of Sweden. The municipality is located on the Baltic Sea but stretches some 50 km into the interior of the country. Härnösand has a history of being the administrative and commercial center as well as the capital of the Region of Västernorrland, which, unlike Härnösand, has a strongly industrial character. In the past, Härnösand was the largest city in the region and long known as the educational and cultural center in the Northern part of Sweden. Today, the population is shrinking, and community leaders are looking for new ways forward to develop the municipality. These pathways include attempts to develop not only sectors such as tourism and the cultural and creative economy but also sustainable food industries. In this study, Härnösand serves as an example of a municipality striving to become more sustainable but has encountered logistical, legislative and hinders for this transition (26). The data gathered for this study consist of a variation of sources from Härnösand, from secondary data on food procurement to interviews with policymakers, all to enable responding to the central research questions guiding this study.

### 2.2 Calculations of the carbon footprint

To measure the carbon footprint (greenhouse gas footprint) caused by food procured in the Härnösand municipality, information about all the food items purchased during 12 months (1^st^ of August 2021 to 31^st^ of July 2022) was extracted from the purchasing system in the municipality (see Appendix). The data contain the type of product, the amount purchased during the period measured in kilograms, address/school, producer, supplier, brand, any sustainability label, and country of origin. The purchase data do not contain the amount of emissions linked to the specific products, and for this purpose, the Food Climate database developed by the Swedish state-owned research institute (RISE) (27) was used. The database includes a carbon footprint based on LCAs of more than 800 (imported and national) food products, representative of Swedish food consumption. The system boundaries used for the LCAs are *cradle to gate* (including all activities starting with the extraction of materials from the earth, their transportation, refining, processing, and fabrication activities until the material or product is ready to leave the process factory or farm). For imported goods, transport to Sweden is included, but packaging and transports within Sweden were excluded. The rationale behind this is that as public procurers buy in bulk and the wholesaler's logistic systems are optimized, packaging and transport constitute a relatively small share of the total carbon footprint and are similar for all product types. In accordance with ISO 14067, the potential changes in soil organic carbon in the agricultural soils are not accounted for in the footprint of the food product.

The carbon footprints provided in the database were calculated based on global warming potential (GWP) retrieved from life cycle assessments (LCA) of food products representative specifically for Swedish food consumption. Climate impact is in all cases expressed in kilograms of carbon dioxide equivalents (CO_2_e) per kilogram of food and thus includes the total climate impact from all greenhouse gases (e.g., carbon dioxide, methane, and nitrous oxide) (27). Using database of RISE, the carbon footprint was supplied for each of the 5,953 products in the purchasing data, multiplied by the total amount of the purchased goods (kg) for the 12 months to obtain the total emissions. The data on the number of goods purchased from the municipality of Härnösand have high accuracy, completeness, and consistency, which can readily be verified. In order to match the 5,953 products with the 800 datasets in the database, assumptions were made. For example, in the purchasing data, 58 types of rice were listed (long grain, short grain, jasmine, basmati, etc.) compared to 11 types of rice in the database. In the case of rice, the matches were based on the country of origin. For sausages, as another example, the carbon footprint of the 44 types listed in the purchasing data was taken from the closest possible match with the 13 types in the database, on the basis of country of origin, meat content, percentage of pork/beef, and whether it was an organic or conventional product. For all vegetables grown in greenhouses (cucumber, tomato, etc.), the assumption was made that all vegetables originating from Holland and Belgium have been grown in greenhouses with a heating source (hence higher emissions) while vegetables originating from Spain were assumed to be grown in open fields or greenhouses without heating (lower emissions). Finally, assumptions about the country of origin were made when the purchased product was not available in the database with the correct country of origin. In those cases, it was assumed that the production was affected by the same emissions, regardless of the production country. Microsoft Excel 2019 was used for these calculations.

### 2.3 Alternative climate mitigation scenarios dietary scenarios

Three alternative scenarios were created. The scenarios were designed with the aim of decreasing the carbon footprint through strategic replacements of the food products associated with the highest emissions according to the carbon footprint calculations by less carbon-intensive alternatives. As strict vegan or vegetarian diets are not readily adopted by the broad public, less carbon-intensive meat sources were included in all scenarios. Essentially, different proportions (in kilograms) of red meat (beef, lamb, pork, and wild meat) were replaced, due to its high carbon footprint, by an equivalent protein source (in kilograms) with a lower carbon footprint. An attempt was made to present beef separately as it is subject to the greatest emissions, but as many semi-finished products contain both beef and pork, these foods were not reported separately.

Scenario 1 was named the “zero red meat scenario.” In this scenario, all red meat was removed from the serving and replaced by vegetable protein. Animal proteins other than red meat (fish & poultry) remained as it was served originally. In scenario 2, the “−50% red meat scenario,” 50% of the red meat was replaced by vegetable protein. Finally, a third scenario was calculated; the “flexitarian free from red meat scenario,” in which all red meat was eliminated, and 50% was replaced by fish and poultry and the other half by vegetable protein.

### 2.4 Obstacles and enablers for decarbonization

To assess the likelihood that a decarbonization of the food procurement system would be carried out, data on obstacles and enablers for a municipality committed to adapting to the necessary changes were collected. To collect this type of information, interviews with the municipal procurement organization, purchasing department, and kitchen in Härnösand were conducted. In addition, suppliers and producers as well as municipalities in other parts of Sweden that have been successful in transforming the procurement procedures were interviewed. By complementing the case study in Härnösand with data from other municipalities that have implemented decarbonization solutions that may be applicable to this case, good examples and potential pathways for improvement in terms of sustainable food procurement were illustrated.

The interview questions were based on earlier studies on food systems and their environmental impact conducted by the research group (28, 29) as well as the few studies in the field that have any type of relation to the specifics of alternative procurement processes (21, 22). However, as there has not been much published on this topic, the interviews were of an exploratory character with a semi-structured interview design. The interviews focused on establishing an understanding of enablers and obstacles for the producers to offer products with a lower carbon footprint and for the municipalities to procure such products. The interviews were carried out by phone during 2022 and 2023, and the transcripts were manually coded and categorized according to four strategic areas that emerged as being the most crucial issues (mentioned by all the respondents): (1) the prevailing Swedish food culture, (2) the role of politicians in the transition, (3) Swedish legislation, and (4) infrastructure. Although the interviews were designed to address decarbonization in general, the respondents tended to focus on increasing the share of local food procurement as the most important measure to achieve decreased GHG emissions. The Food Climate Database shows that Swedish meat (and to a lesser degree also Swedish vegetables) is typically associated with a lower carbon footprint due to different production methods and shorter transports (27).

Despite a municipal policy to support local food production, most of the purchased food in Härnösand is imported from other Swedish regions or abroad. Five local food producers were thus selected representing the three most common food categories produced in the region: vegetables, meat, and potatoes. They were interviewed about perceived obstacles and enablers to deliver food to the municipality. Attempts were made to identify producers who conduct their business within Härnösand immediate area, but as that was not entirely possible, producers were recruited from geographically adjacent regions. The interviewed producers were as follows:

- Producer 1, potato producer in Härnösand.
- Producer 2, beef producer in Stöde/Sundsvall.
- Producer 3, beef producer in Kovland/Sundsvall.
- Producer 4, vegetable producer in Härnösand.
- Producer 5, potato producer, Östersund.

A part from the case municipality Härnösand, four other municipalities that have documented accomplishments within sustainable food procurement (30) were selected to be interviewed. Those four municipalities were selected from “Ekomatligan,” the top-ranking Swedish municipalities based on their certified organic purchases. Municipalities of a size comparable to Härnösand with documented examples of creative and innovative ways to carry out their purchases, i.e., buying cows, instead of processed beef meat, were selected. The food and service managers and directors of nutrition were interviewed from one and each of the four municipalities. Illustrative quotes from the interviewees were selected aiming to represent the view of all interviewed municipalities. The interviewees received full disclosure of the study objective, all interviews were carried out with informed consent, and the transcripts were anonymized. The research is not subject to the Swedish Ethics Review Act (2003:460) as it does not handle sensitive personal data according to the data protection regulation (GDPR) of EU or aims to influence research subjects physically or psychologically.

The aim of these interviews was to identify success factors for transforming the PFP toward sustainability and decreased GHG emissions. There is a public register in Sweden of GHG emissions associated with food in the public sector (the lack of synthesized open-access LCA data on food in the public domain is a limitation, indicated by, e.g., Clune, Crossin, and Verghese (14), that limits informed decision-making). However, there are statistics on the proportion of Swedish and organic food that are purchased within Swedish municipalities. In this study, it has been assumed that the municipalities that make an extra effort to procure organic and Swedish products may also be eager to promote decarbonization or other aspects of sustainability. Municipalities that have a high proportion of organic purchases were thus defined as “good examples.”

## 3 Results

The results from carbon footprint analysis show that the emissions in the municipality of Härnösand need to, and can be, reduced in order to comply with the Paris agreement and the municipal policies.

### 3.1 The total carbon footprint

For the investigated 12-month period, the GHG emissions associated with the food service in the municipality of Härnösand deviated from the global sustainability goals. During the period 1^st^ of August 2021 to the 31^st^ of July 2022, the GHG emissions were on average 2.2 kg CO_2_e per kilo of food served. To comply with the municipal sustainability goals (that are based on the Paris Agreement), the GHG emissions associated with the food service need to be reduced by 40.9% to 1.3 kg CO_2_e per kilo of food served.

**Figure 1:**
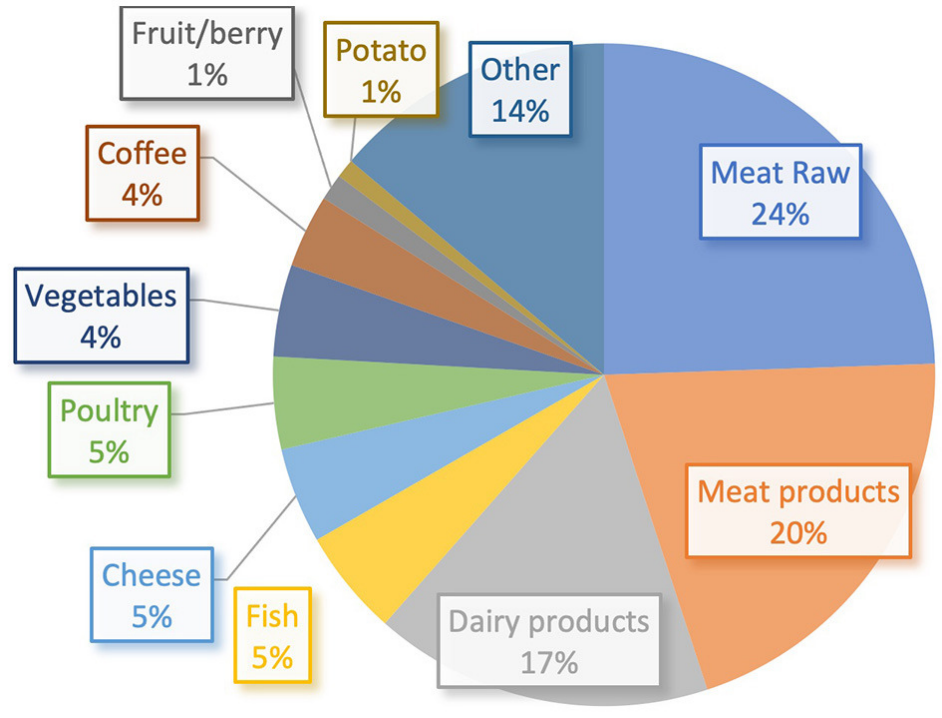
Share of GHG emissions per food category.

Figure 1 illustrates how the emissions correlate to the respective food category: 76% of the emissions derive from food of animal origin: 44% of the emissions are derived from processed or unprocessed red meat (pork, lamb, wild meat, or beef).

**Figure 2:**
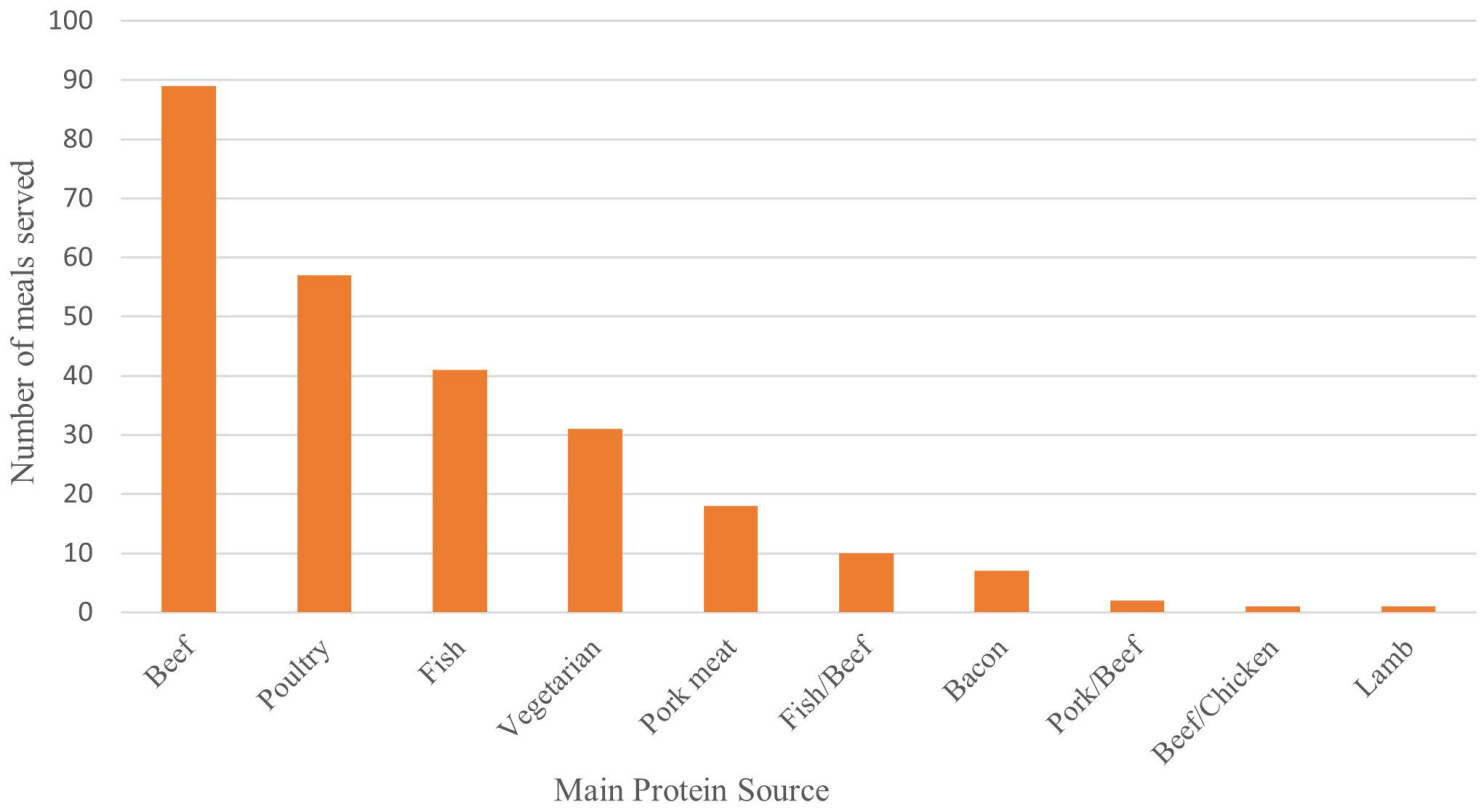
Main protein source per served meal.

Figure 2 displays the origin of the main protein source served at each meal during the 12-month period. The proportion of pork that is bacon was large and thus reported separately (28%). When the 31 vegetarian meals were examined, it was revealed that half of them were pancakes.

### 3.2 Redistribution of the food procurement

**Figure 3:**
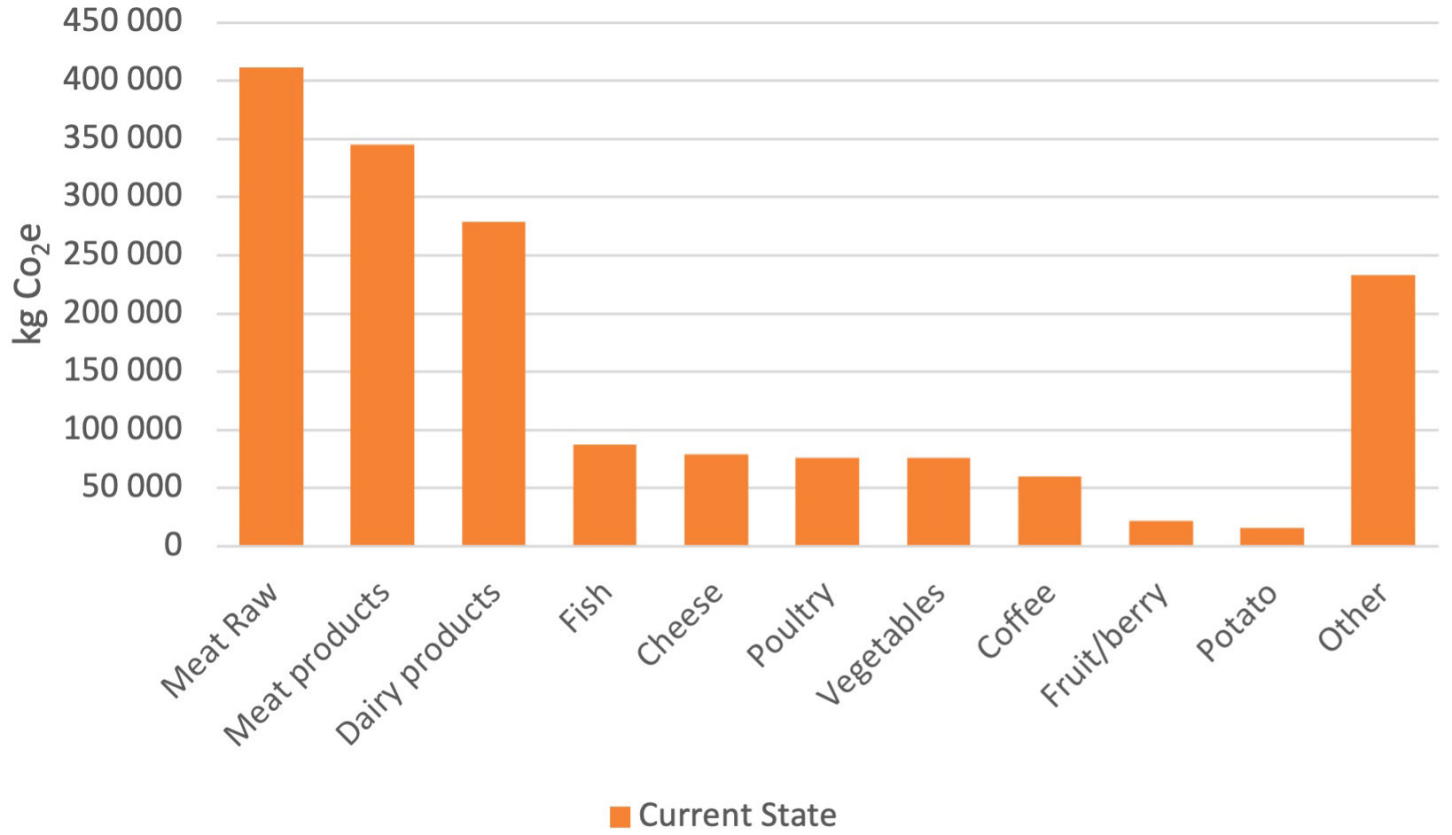
Current state of total CO_2_e emissions (kg) per food category.

The carbon footprint from the three alternative scenarios compared to the baseline (current state) is shown in Figure 3. The carbon footprint can be reduced by 44% if Härnösand municipality chooses to adopt the “Zero red meat” scenario, by 22% if the “−50% red meat scenario” is chosen, and finally by 39% if the “flexitarian free from red meat” scenario is chosen. It is noteworthy that the fractions that were altered in the three scenarios (meat, vegetable protein, and fish/poultry) constituted only 6% of the total purchased food in terms of mass (in kilograms). However, the cutback of meat caused GHG emissions reductions as high as 44%.

The orange bar illustrates today's emissions, 1,682,148 kg CO_2_e for the 12-month period. During the same period, 762,391 kg of food was purchased, which generated 2.2 kg CO_2_e per kilogram of food served. To reach its goals of reducing GHG emissions to a level in accordance with the Paris Agreement, the municipality of Härnösand could adopt either the “flexitarian free from red meat” (1,35 kg CO_2_e per kilo served food) or the “zero red meat scenario” (1,24 kg CO_2_e per kilo served food) by 2030.

**Figure 4:**
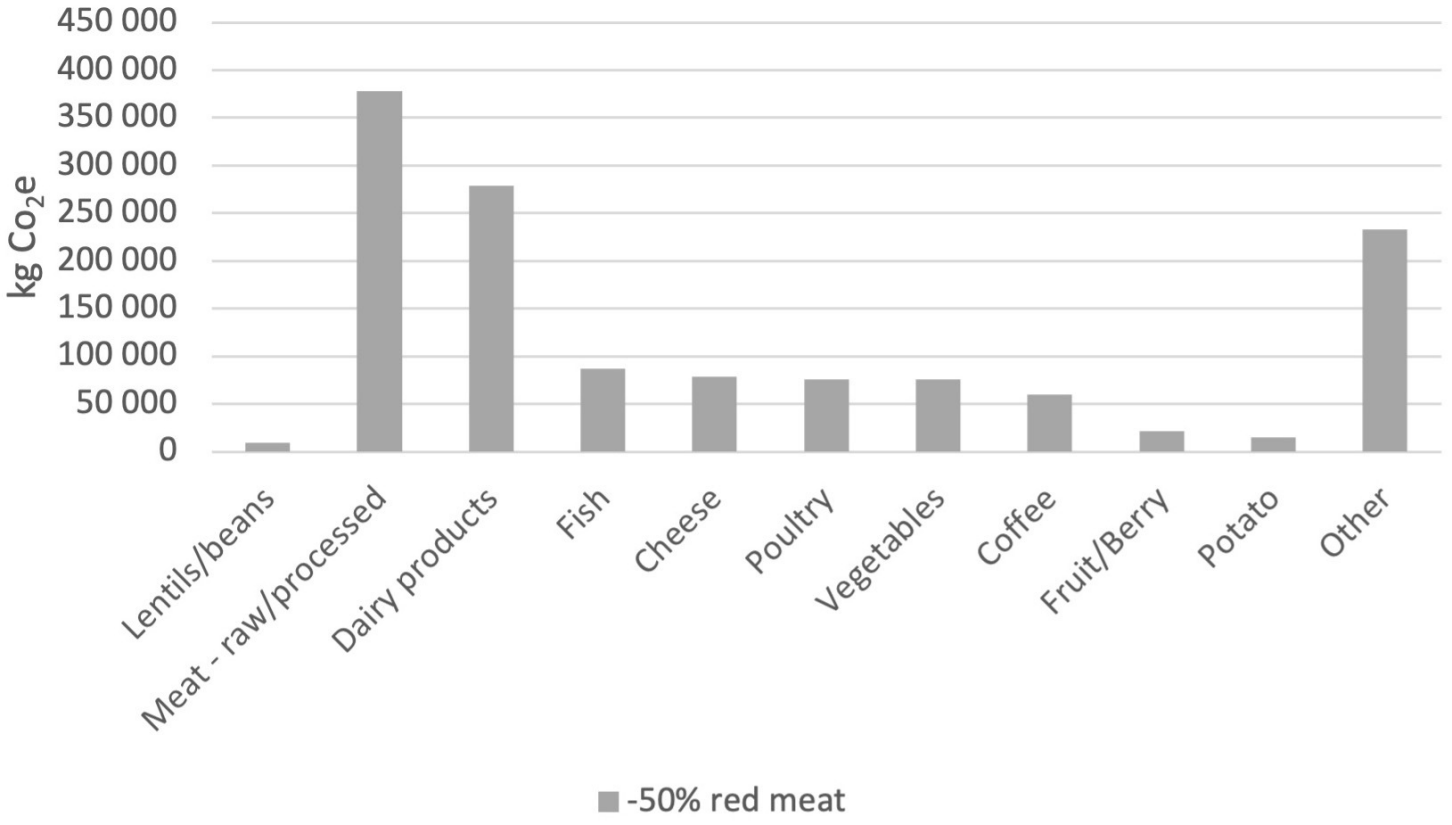
Total CO_2_e emissions (kg) per food category from the −50% red meat scenario.

**Figure 5:**
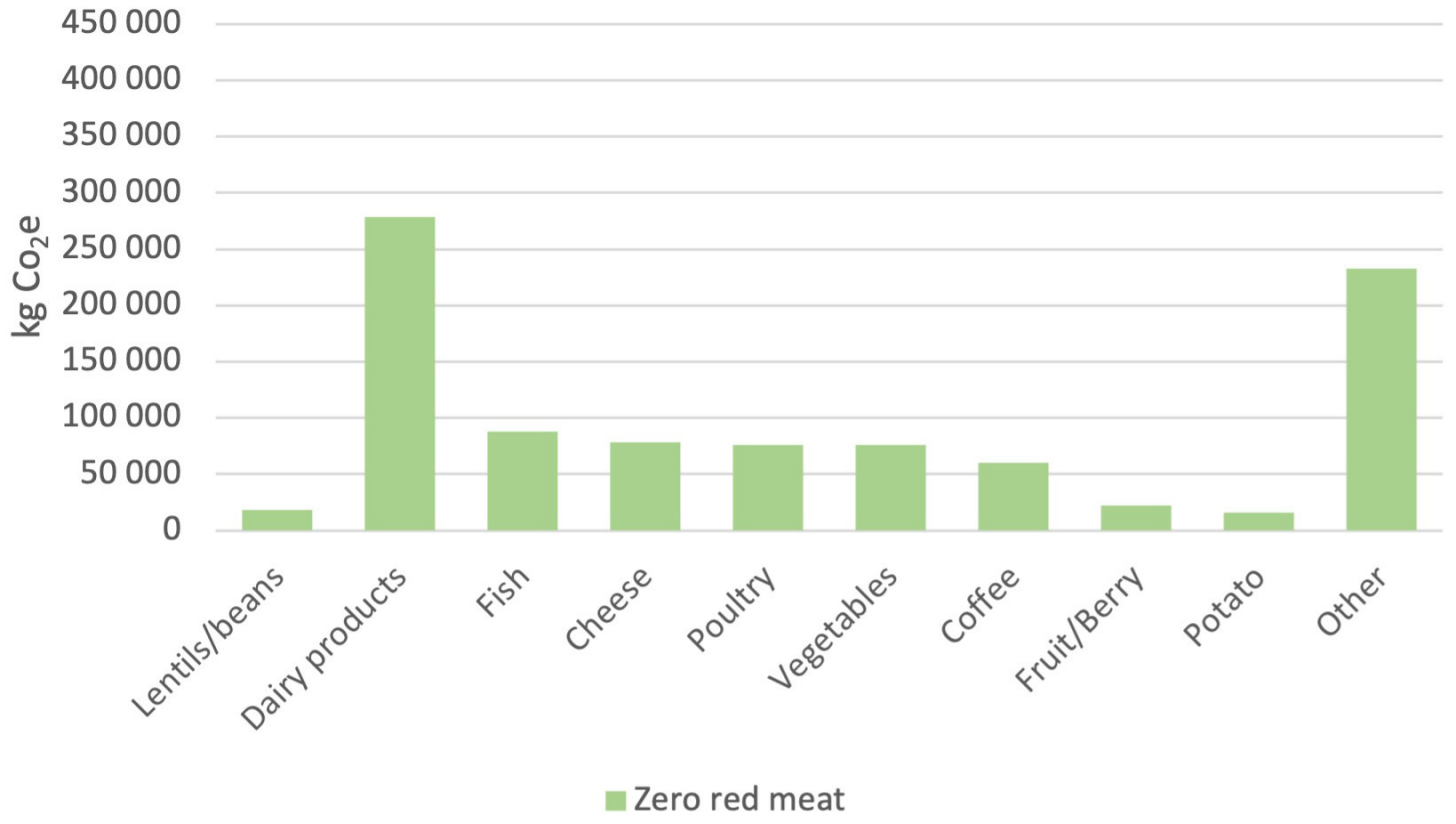
Total CO_2_e emissions (kg) per food category from the zero red meat scenario.

**Figure 6:**
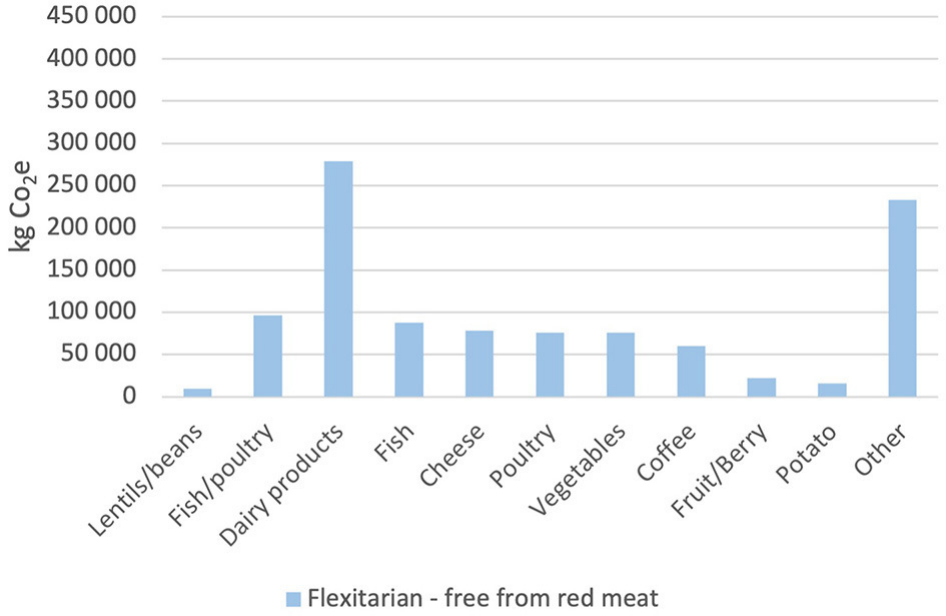
Total CO_2_e emissions (kg) per food category from the flexitarian free from red meat scenario.

Figures 4–6 show the effect of replacing red meat as food with alternative protein sources. The y-axis shows the kilograms of CO_2_e that the specific food group has emitted during the 12-month period. Raw meat and processed meat are together emitting 411,542 and 344,555 kg CO_2_e, respectively, during that period. Replacing all red meat with vegetable protein, the “zero red meat” scenario would entail a reduction in emissions by 756,097 kg CO_2_e per year, which is a reduction of ~45%. The −50% red beef scenario renders a decrease of 22% and a total emission of 1,313,299 kg CO_2_e for the period. The emissions from legumes end up at 9,199 kg CO_2_e, while the emissions from the red meat are 378,049 kg CO_2_e. The “flexitarian free from red meat” scenario is the closest to what is required of the municipality to reach the Paris Agreement. It renders a decrease in emissions at 39% where the legumes emit 9,199 kg CO_2_e, and the mix of fish and poultry emit 96,300 kg CO_2_e.

### 3.3 Obstacles and enablers for the decarbonization of the municipal food procurement

This section is derived from interviews with producers, successful municipalities, and the municipality of Härnösand. The respondents mentioned several areas where transformation was determined necessary and where transformation was structurally hampered, henceforth referred to as strategic areas, where interventions are necessary to catalyze change. They also mentioned creative solutions to circumvent this obstacle and catalyze change. Four strategic areas stood out as they were mentioned by all the respondents: (1) the prevailing Swedish food culture, (2) the role of politicians in the transition, (3) Swedish legislation, and (4) infrastructure (see Table 1).

**Table 1:** Perceived obstacles and enablers by producers, other interviewed municipalities, and the procurement department in Härnösand.

**Strategic area**	**Obstacles**	**Enablers**
The prevailing Swedish food culture:	• The difficulty of promoting a new food culture to children and the elderly. • Reluctance to changing eating habits from, e.g., cucumbers and tomatoes to cauliflower. • The change of food culture is typically a slow process. • Tradeoffs between introducing new foods and the national goal of no food waste.	• Educating children, staff, and parents provides understanding and acceptance of new foods. • National goals and guidelines can speed up cultural change. • Moving the responsibility for changing food culture one level up from public kitchens to politicians and decision-makers.
The role of politicians in the transition:	• Lack of or unspecific sustainability goals from the national level places the responsibility for sustainability work on municipalities. • Legislative ambiguity—laws are interpreted by each municipality individually creating uncertainty and hampering change. • In a small municipality with a tight budget there is a risk that decarbonization is not prioritized.	• Political engagement, unity, and long-term perspective at the local level. • Clearly communicated policies in terms of food procurement and a pertinent budget for that policy to be implemented.
Swedish legislation:	• A high level of competence in Swedish laws and regulations is needed for employees in public kitchens, to be able to structure the procurement appropriately. • Swedish legislation favors large wholesalers and smaller local food producers have a disadvantage. • Rigid legislation. • Conflict of aims between the Swedish Public Procurement Act and other Swedish policies (food strategy; sustainability goals).	• Intermediaries can make procurement easier for small producers as small-scale producers often decline to participate in public procurement due to the workload. • National legislation and policy instruments may make sustainable food transformation legally binding.
Infrastructure:	• Kitchen with inadequate equipment to handle unwashed vegetables, uncut meat, and bigger volumes of unprocessed food. • Low staff density forces the kitchens to purchase processed food that does not require so many work steps. • It is often the producer's responsibility to deliver to all the units of municipality, which puts a large responsibility on small producers.	• Kitchen adapted to manage unprocessed foodstuffs. • Increasing the number of staff to have flexibility in the kitchen and to be able to receive raw materials that are not fully processed gives more opportunity to choose smaller local suppliers. • Coordinated goods distribution, where the municipality has a delivery method for all goods and ensures that it is then distributed to the respective unit.

#### 3.3.1 The prevailing Swedish food culture

All the interviewed kitchens mentioned the challenge of getting children and the elderly to be curious about new diets. An interviewee from one of the interviewed municipalities said,

“We are working towards bringing in more plant-based food—it's a slow process. It is a challenge; we are working actively towards the vegetarian dishes; the children are slowly beginning to recognize and enjoy them. We are a ‘workers town,' where potatoes and meat have been a big part of our food culture for a long time. We are serving parallel dishes that are vegetarian, still, it is difficult to make the transition to plant-based food. Nevertheless, the children are choosing something else.” (Food and service manager, municipality 1)

Many respondents claimed that it is a difficult task for a single school kitchen to educate about food literacy and break prevailing norms. Replacing animal protein with plant-based protein was mentioned as a particularly cumbersome challenge. Several kitchens mentioned heavy resistance from students, parents, and even the municipal management itself.

#### 3.3.2 The role of politicians in the transition

All the interviewed kitchens and producers stressed the importance of committed local politicians. Representatives from the successful municipalities said that the politicians' clarity and long-term perspective have been decisive. The two municipalities that have not succeeded in the shift to a menu with a lower carbon footprint both claimed that the absence of goals, governance, and support from politicians means that the responsibility of transition ends up in the hands of individual procurers and buyers. Sweden is a decentralized country where municipalities have a large possibility for self-determination. How the municipalities choose to implement the Paris Agreement is therefore highly varying. The interviewed kitchens and purchasing organizations all claim that it is partly difficult to interpret the implications of the Paris Agreement for their organization. It was also highlighted that that the study toward a decarbonized food system largely depends on the willingness of the ruling politicians and their attitude toward sustainability issues. One of the interviewed municipalities had no sustainability goals at all; hence, the kitchen staff tried to implement sustainability goals voluntarily, in addition to fulfilling the goal of municipality, which was to buy local food.

“We live in a ‘meat-municipality.' If we look at the global goals and raise them within the municipality board, it will not be well received. So, we try to work on this ourselves, to bring in more vegetables.” (Director of Nutrition, municipality 4)

#### 3.3.3 Swedish legislation

Swedish legislation is often perceived as an obstacle by procurers, producers, retailers, etc., who aspire to promote decarbonization in the local food systems. It was a general opinion among the interviewees that Swedish legislation makes local purchases difficult or even impossible. Producers and successful municipalities alike expressed that they believed that the reason they succeed in buying/selling; e.g., local food is because they have decided to bypass the Swedish Law on Public Procurement (LOU). Bypassing the law may mean buying living animals, instead of meat, owning a high school that produces its own food for the municipality, or being more involved in the procurement than what is typical. Municipality 3 have introduced a completely new procurement procedure to be able to purchase locally produced meat for its kitchens. Instead of buying processed meat, they buy cows. In March 2022, the municipality bought 24 cows whose meat will eventually be consumed in the elderly care and schools. In all phases, from the procurer to the kitchen and the producer, different kinds of frustrations were expressed. One producer stated that:

“The legislation concerning public procurement discourages all forms of small producers, local producers. The legislation goes against the entire Swedish production because Swedish farmers and producers must compete with imported products that we would have been jailed if we had produced in the same way.” (Business owner, Producer 2)

One head chef said,

“On a national level, the most important thing we can do is promote changes in the procurement policy. We need new legislation that allows a different type of purchasing. We take risks to be able to make the purchases we do.” (Food- and service manager, Municipality 3)

And one procurer claimed,

“The Swedish Law on Public Procurement (LOU) and Swedish food strategy counteract each other.” (Business owner, Producer 4)

In Municipality 4, they have been implementing a project since 2005 called “reclaimed” which is a political endeavor that aims to bring back local agriculture, food, and open landscapes. They also own their high school for Natural resources where the students, among other things, produce meat and vegetables and the municipality does not have to take LOU into account for food delivered from that school. Notwithstanding, representatives from Municipality 4 mentioned LOU to be an impediment to procuring food outside of their own production.

“The Swedish Law on Public Procurement (LOU) makes it difficult for us. We cannot say that we want local vegetables or meat, you must set the requirements according to what you want, and the preparatory work is extremely important, a good dialogue with the producers so that you succeed in finding the specific uniqueness that allows us to make the right demands and that we get exactly the local product we want.” (Director of Nutrition, Municipality 4)

Municipality 1 have procured Highland Cattle with the purpose to maintain meadow ecosystems, in the autumn, the animals can be slaughtered and delivered to local kitchens without breaking the LOU. They expressed that the key was to purchase their own animals for another purpose than food. One meat producer said,

“If the procuring municipalities had followed laws and regulations to 100%, we would not have been able to participate in the procurement process. The municipality and the politicians' willingness to invest in local food has been decisive for us as suppliers.” (Business owner, Producer 2)

And a producer of vegetables stated,

“The biggest problem is that in public procurement, it is allowed to buy in products from abroad that are not produced in accordance with Swedish legislation. (—) Why is it allowed to import goods today that we can produce in Sweden? We still have other rules for the use of chemicals in Sweden, which we must produce at the same price as in other countries.” (Business owner, Producer 5)

#### 3.3.4 Infrastructure

The last strategic area was infrastructure. The possibilities to store, transport, receive, and process food were mentioned as a prerequisite for a decarbonized procurement and the absence of one part of that chain may be a serious obstacle. Today, according to both the producers and the kitchens, the responsibility of the entire food handling infrastructure is largely transferred to producers and individual kitchens. All interviewees expressed, in different terms, that this is an unreasonable burden that is also ineffective and very costly for society. All the successful municipalities mentioned that they have their own transport/storage infrastructure in place and that this has been significant to be able to procure food outside the big distributors.

A potato producer who was interviewed said that the prerequisite for them to be able to sign up as a supplier is that they manage the deliveries themselves to each address in the municipality. In their case, that means that they need to deliver potatoes to 70 different addresses which entails a large overhead cost that needs to be applied to only one product in competition with the large distributors who spread these costs over hundreds of goods. The municipalities that have been successful in bringing in local smaller producers have all chosen to invest in a municipal coordinated goods collection center. In such a center, all suppliers deliver to one address and the municipality itself manages the distribution to schools and homes for the elderly. Several schools mentioned that the collection center, in addition to the environmental- and cost-benefit, also contributed to increased safety and a sounder work environment due to fewer means of transport to areas where there are children and less stress for employees.

Many kitchens also expressed that their ability to receive and handle alternative food has been greatly hampered by the fact that many kitchens today are only heating kitchens and lack cooking facilities. In such a kitchen, the staff is limited to receiving pre-cooked food. The responsibility to use ingredients with a low carbon footprint is thus moved from the kitchen to the distributors. Staff density is also a limitation; one kitchen testified that they can only receive vacuum-packed minced meat, as all other raw meat materials involve work that they do not have time for. The same applies to vegetables; only processed vegetables can be handled due to lack of time in many kitchens.

One producer explained,

“The specialization in public kitchens makes it difficult for “ordinary” producers to deliver their food as they are rarely refined to a sufficiently high extent. For example, you can rarely sell unpeeled potatoes, parts of meat that are not minced meat, vegetables that require processing (chopped, etc.).” (Business owner, Producer 3)

One of the successful municipalities (Municipality 2) has made extensive changes in its kitchens to increase their opportunities to make use of whole foods and reduce waste. As for the premises, they have been adapted so that larger kitchens have been given meat grinders and bread-slicing machines. Then, over the years, when equipping kitchens, they have chosen to rebuild the heating kitchen into a cooking kitchen. The same applies to new buildings. Currently, the municipality mostly have cooking kitchens, and to make good use of the food waste, they have invested in more cooling cabinets.

## 4 Discussion

Decreasing the carbon footprint of the food system is a complicated procedure incorporating economic, environmental, and social dimensions that must be integrated coherently to be successful. The results from the carbon footprint analysis show that the GHG emissions associated with the current food service in Härnösand need to be reduced by 41% to comply with the sustainability goals of the municipality (based on the Paris Agreement); 76% of the GHG emissions from the food served in the municipality derive from animal food sources (although they only constitute 40% of purchased food kilograms). This is in accordance with, e.g., Sandström et al. (11) who demonstrated that over 80% of the GHG emissions of the European Union diets are caused by the consumption of meat, dairy, and eggs. Unprocessed red meat was responsible for 44% of the total GHG emissions in Härnösand although they only constitute 6% of purchased food kilograms, suggesting that a reduction in servings of red meat would be an appropriate way to start the transition toward a decarbonized diet. It is noteworthy that none of the two alternative climate mitigation scenarios that were compatible with the goals of the Paris Agreement (“flexitarian free from red meat” and “zero red meat scenario”) are strictly vegetarian. Rather, they are flexitarian diets without red meat. If Härnösand were to stop serving red meat, emissions would decrease significantly but as a single school or local area departing from the current societal norm around food, such a measure would be difficult to implement. There are examples of municipalities that have tried to serve more plant-based food but have been forced to back down due to pressure from students and parents. Härnösand (among many other municipalities in Sweden) offers a plant-based option to its students every day. However, the students rarely chose that option with increased food waste as a potential consequence.

While the results concerning the emissions support the existing knowledge on emissions from food, the data from the interviews provide some unique and site-specific testimony of the difficulties that many municipalities face in complying with national and international sustainability goals within the boundaries set by budgetary restriction, procurement legislation and prevailing conditions in kitchens and among producers. The region where Härnösand is located is sparsely populated, which may make some of the outcomes less applicable in more densely populated areas. The findings do not necessarily reflect the diversity of opinions and experiences that would have been found if the sample had been chosen from all regions in Sweden. However, the picture that emerges from interviewing the different actors in the local food system suggests that the transition toward a decarbonized food system is a national concern that many municipalities are trying to solve locally. However, as the food systems are partly national and many times global, it is difficult for small regions to bring about change without national coordination and overall collaboration. Nevertheless, experiences from Portugal suggest that successful examples from small towns or cities can play a key role in fostering resilient and economically prosperous food systems given their proximity and close interaction with relevant economic and societal actors (31).

While it can be argued that the responsibility for the transition toward a decarbonized food system, in the public sector, lies with each and every one of the individual kitchens, the interviewees from kitchens, procurement offices, producers, and successful municipalities, all claim that the herculean efforts that such a transformation entail, need to be coordinated from a higher organizational level to be effective. The interactional dynamics among the actors involved in food procurement are often highlighted as an important factor that defines the successful implementation of sustainable food procurement (32, 33).

Many municipalities mention that they are struggling to reach the national and global sustainability goals and are aware of the importance of changing the local food service to succeed in this. It should be noted, however, that many respondents focused on the importance of increasing local food procurement as the most efficient and feasible measure to decrease GHG emissions. This may be due to the widespread belief that local food is analogous to sustainable food (34, 35). This simplified look at sustainable PFP seems to be structural, and Molin et al. conclude that the scientific literature is strongly oriented toward locally sourced and organic foods overlooking other aspects of sustainability (21). While the Food Climate Database suggests that local and national meat and some vegetables are associated with a lower carbon footprint, the transformation in the food necessary to comply with the Paris Climate Agreement goes well beyond just increasing the share of locally produced food. Smaller regions have limited possibilities to catalyze the change alone. The national guidelines that would encourage other actors (wholesalers, producers, etc.) in the food chain to facilitate the transformation toward a more sustainable food system were deemed largely insufficient by the respondents in this study. Galli et al. stress (31) the importance of proactive communication among various government levels and various actors, and a clear alignment of interests and strategies from the national toward the local levels in order to achieve local disruptions toward sustainable food transition.

Some of the obstacles mentioned above can be circumvented through creativity, by stretching regulations without the breaking law and by increasing the number of kitchen staff. However, most municipalities express that they are not entirely free to purchase food based on decarbonization criteria and call for more flexible legislation. The Swedish Law on Public Procurement (LOU), deficient infrastructure, an unsustainable food culture, and local politicians' reluctance to change were mentioned as the main obstacles. The interview data suggest that to align the Swedish food system with the Paris Agreement and the Sustainable Development Goals, national and regional policies need to be updated to address these obstacles. This is in accordance with Mattas et al. who conclude that the PFP requires coherent and coordinated European and national policies and highlights the insufficiently designed and adapted regulatory framework as an impediment to successful PFP (36) and Smith et al. (37) who call for operational policies for the food procurement sector.

Although Sweden is among the few countries that have mentioned climate considerations and the relative impact of various food groups in its national dietary guidelines (NDG) (2), the Swedish NDGs do not consider the environmental impacts in their dietary recommendations. Swedish politicians are often reluctant to take a stand on the issue of dietary changes. In 2016, Sweden's then Minister of Rural Development said,

“I do not want to police what should be on the Swedes' breakfast or dinner table” and added that: “Swedish politics should stay away from the Swedish kitchen.”

Sven-Erik Bucht, Minister of Rural Development 2016 (38).

This view on Swedish eating habits seems to endure among the first-level executive bodies. During the revision of the Swedish current food strategy, Sweden's current Minister of Rural Development claimed,

“I do not intend to interfere with people's eating habits, if you eat meat once a week or every day.”

Peter Kullgren, Minister of Rural Development 2023 (39).

Without operational national goals in terms of how to catalyze the necessary changes in the food system (including dietary changes), it seems unrealistic to expect that municipalities will bring about the required transformation based on voluntary commitments and within the current budgets. Without a national common stance on how to transform the food system, the progression toward diets with carbon footprints in line with the commitments of the Paris Agreement is likely to be slow.

The Swedish National dietary guidelines regarding meat endorse consumers should eat less red meat and cured meats—preferably < 500 g per week. In the planetary health diet proposed by the EAT-Lancet Commission, the amount of intake of red meat for an adult is 98 g/week (2). The Swedish Agricultural Agency states that the consumption by 2019 was 28.5 kg/person and year (548 g/week) according to the 2019 figures. The consumption of red meat, on average, in Sweden today is approximately 10 % above the Swedish dietary recommendations, and hence considerably higher than the planetary health diet. According to the Swedish Environmental Protection Agency, each Swede cause, on average, approximately 1.5 metric tons of CO_2_e per person and year by just eating (40). The Nordic Food Policy Lab (financed by the Nordic Council of Ministers and the health and food authorities in the Nordic countries), released the fifth edition of its Nutrition Recommendations in June 2023. The Nordic Nutrition Recommendations constitute the scientific basis for food-based dietary guidelines in Denmark, Finland, Iceland, Norway, and Sweden. The fifth edition (NNR 2022) that integrates climate and environment discourages eating more than 350 g of red meat/per person/week for health reasons and preferably lower than that to decrease the environmental impact. It is yet to be seen how the Nordic recommendations affect the Swedish Dietary Guidelines. One of the conclusions of Willet et al. (2) is that it is impossible to change our food systems, unless citizens change their approach to consumption, cooking, and handling of food. While people's preferences are largely driving the transformation of the food system, such preferences are strongly influenced by marketing, National and international policies, and advocacy work of lobbyist organizations. Hoek et al. (8) claim that social norms have a great influence on people's decision-making and public procurement holds a significant potential to shape norms around food (9). Change agents and policymakers may promote individual decisions and behaviors toward a more sustainable food system, for example, changes in food production practices or a radical dietary change.

One argument that often recurs in municipal operations is insufficient funding. Public kitchens, just like any other municipal organization, have limited money to spend, and more sustainable alternatives are often associated with high costs. However, Municipality 3 testifies that their costs have not increased, due to the fact that they use all parts from the animals in their cooking. An interesting continuation of this project would be to study the social benefits of increasing local production, instead of assuming that sustainable food is associated with high costs, could there be benefits to reshaping the food systems, becoming more local, eating less and better meat? How many jobs could potentially be created? What tax revenue would it generate? Are there potential health benefits? Moreover, is it possible to estimate a scenario where the municipality of Härnösand would purchase, e.g., 40% of its food from local suppliers? These are questions that such a study could address.

It should be noted that, in this study, only the environmental impact of GHG emissions was considered. A sustainable diet cannot be reduced to one that has a low carbon footprint. It also needs to be protective and respectful of biodiversity and ecosystems, culturally acceptable, accessible, economically fair, and affordable; nutritionally adequate, safe, and healthy; while optimizing natural and human resources (41). Tradeoffs and conflicts of aim between different environmental goals need to be considered in any policy that aims to transform the food system. Härnösand, which is a medium-sized municipality with relatively few inhabitants, has proven to have additional challenges that are partly due to its geographical location. The municipality is located in the Northern half of Sweden with a climate that does not allow the same number of harvests per year or that the same crops can be grown as in the agriculturally more productive southern Sweden. In its attempts to procure local and locally produced food, the biggest difficulty for Härnösand has been finding local suppliers. They simply do not exist. However, interview data from respondents participating in the Food for Life program in the UK show that public procurement can increase the demand for local, sustainably produced food (22) and thus create incentives for new establishments in the food production sector.

## 5 Conclusion

Considering that the current global food system is the largest pressure caused by humans on the earth's ecosystems and that up to 34% of our GHG emissions today can be traced to our food systems (primarily to the food of animal origin), dietary changes are an efficient way to reduce negative environmental impact. The public sector holds a strong transformative power, in terms of catalyzing this change and this study points out some alternative scenarios for the menu composition that would considerably decrease its currently high carbon footprint. This study shows that changing the procurement processes by altering 6% of the total purchased food kilograms. GHG emissions reductions as high as 44% could be obtained. There are many obstacles for such a change in the menu composition to materialize. The results indicate that the Swedish Law on Public Procurement (LOU), deficient infrastructure, an unsustainable food culture, and local politicians' reluctance to change are the principal impediments perceived by the interviewees.

The respondents stressed that the policy change in Sweden needs to be coordinated from a higher organizational level but that there are also opportunities for individual municipalities to take certain measures. Political decisions about the decarbonization of the food systems, willingness to invest in logistics centers, or other ways to help solve the issue of food logistics were highlighted as important first steps. To explore the issue of promoting public procurement as a lever for changes in the food system, further studies on National food policy issues, sustainable food leadership, and the social benefits of increasing local production in Sweden are recommended.

## Data Availability

The raw data supporting the conclusions of this article will be made available by the authors, without undue reservation.

## References

[1] Rockström J Gaffney O Rogelj J Meinshausen M Nakicenovic N Schellnhuber HJ. A roadmap for rapid decarbonization. Science. (2017) 355:1269–71. 10.1126/science.aah3443 28336628

[2] Willett W Rockström J Loken B Springmann M Lang T Vermeulen S. Food in the Anthropocene: the EAT–Lancet Commission on healthy diets from sustainable food systems. Lancet. (2019) 393:447–92. 10.1016/S0140-6736(18)31788-4 30660336

[3] Crippa M Solazzo E Guizzardi D Monforti-Ferrario F Tubiello FN Leip A. Food systems are responsible for a third of global anthropogenic GHG emissions. Nat Food. (2021) 2:198–209. 10.1038/s43016-021-00225-9 37117443

[4] Searchinger T Waite R Hanson C Ranganathan J Dumas P Matthews E. Creating a sustainable food future: a menu of solutions to feed nearly 10 billion people by 2050. Final report. WRI (2019).

[5] Theurl MC Lauk C Kalt G Mayer A Kaltenegger K Morais TG. Food systems in a zero-deforestation world: dietary change is more important than intensification for climate targets in 2050. Sci Total Environ. (2020) 735:139353. 10.1016/j.scitotenv.2020.139353 32474248

[6] Herrero M Thornton PK Mason-D'Croz D Palmer J Benton TG Bodirsky BL. Innovation can accelerate the transition towards a sustainable food system. Nat Food. (2020) 1:266–272. 10.1038/s43016-020-0074-1

[7] Rockström J Edenhofer O Gaertner J DeClerck F. Planet-proofing the global food system. Nat Food. (2020) 1:3–5. 10.1038/s43016-019-0010-4

[8] Hoek AC Malekpour S Raven R Court E Byrne E. Towards environmentally sustainable food systems: decision-making factors in sustainable food production and consumption. Sustain Product Consumpt. (2021) 26:610–26. 10.1016/j.spc.2020.12.009

[9] Swensson LFJ Hunter D Schneider S Tartanac F. Public food procurement as a game changer for food system transformation. Lancet Planetary Health. (2021) 5:e495–6. 10.1016/S2542-5196(21)00176-5 34390660

[10] Rocha CF Silva CD Silva RM Oliveira MJ Neto BD. The dietary carbon footprint of portuguese adults: defining and assessing mitigation scenarios for greenhouse gas emissions. Sustainability. (2023) 15:5278. 10.3390/su15065278

[11] Sandström V Valin H Krisztin T Havlík P Herrero M Kastner T. The role of trade in the greenhouse gas footprints of EU diets. Global Food Secur. (2018) 19:48–55. 10.1016/j.gfs.2018.08.007

[12] Resare Sahlin K Röös E Gordon LJ. ‘Less but better' meat is a sustainability message in need of clarity. Nature Food. (2020) 1:520–2. 10.1038/s43016-020-00140-5 37128007

[13] Röös E BajŽelj B Smith P Patel M Little D Garnett T. Greedy or needy? Land use and climate impacts of food in 2050 under different livestock futures. Global Environ Change. (2017) 47:1–2. 10.1016/j.gloenvcha.2017.09.001

[14] Clune S Crossin E Verghese K. Systematic review of greenhouse gas emissions for different fresh food categories. J Clean Prod. (2017) 140:766–83. 10.1016/j.jclepro.2016.04.082

[15] Kwasny T Dobernig K Riefler P. Towards reduced meat consumption: a systematic literature review of intervention effectiveness, 2001–2019. Appetite. (2022) 168:105739. 10.1016/j.appet.2021.105739 34648912

[16] Conijn JG Bindraban PS Schröder JJ Jongschaap RE. Can our global food system meet food demand within planetary boundaries? Agric Ecosyst Environ. (2018) 251:244–56. 10.1016/j.agee.2017.06.00134312092

[17] Springmann M Clark M Mason-D'Croz D Wiebe K Bodirsky BL Lassaletta L. Options for keeping the food system within environmental limits. Nature. (2018) 562:519–25. 10.1038/s41586-018-0594-0 30305731

[18] Steffen W Richardson K Rockström J Cornell SE Fetzer I Bennett EM. Planetary boundaries: guiding human development on a changing planet. Science. (2015) 347:1259855. 10.1126/science.1259855 25592418

[19] Röös E Patel M Spångberg J Carlsson G Rydhmer L. Limiting livestock production to pasture and by-products in a search for sustainable diets. Food Policy. (2016) 58:1–3. 10.1016/j.foodpol.2015.10.008

[20] Swensson LFJ Tartanac F. Public food procurement for sustainable diets and food systems: The role of the regulatory framework. Global Food Security. (2020) 25:100366. 10.1016/j.gfs.2020.100366

[21] Molin E Martin M Björklund A. Addressing Sustainability within Public Procurement of Food: A Systematic Literature Review. Sustainability. (2021) 13:13395. 10.3390/su13231339532912299

[22] Morley A. Procuring for change: an exploration of the innovation potential of sustainable food procurement. J Clean Prod. (2021) 279:123410. 10.1016/j.jclepro.2020.123410

[23] Morgan K Sonnino R. The school food revolution: public food and the challenge of sustainable development. London: Routledge. (2013). 10.4324/9781849773256

[24] Olsson D Öjehag-Pettersson A. Buying a sustainable society: the case of public procurement in Sweden. Local Environ. (2020) 25:681–96. 10.1080/13549839.2020.1820471

[25] Konkurrensverket and Upphandlingsmyndogheten Statistik om offentlig upphandling 2020 A. Toyrä, Editor. 2020: Kalmar.

[26] Haller H Fagerholm AS Carlsson P Skoglund W van den Brink P Danielski I. Towards a resilient and resource-efficient local food system based on industrial symbiosis in härnösand: a Swedish case study. Sustainability. (2022) 14:2197. 10.3390/su14042197

[27] RISE RISE food climate database. (2023) RISE.

[28] Nicolosi A Laganà VR Laven D Marcianò C Skoglund W. Consumer habits of local food: Perspectives from northern Sweden. Sustainability. (2019) 11:6715. 10.3390/su11236715

[29] Rytkönen PI Skoglund W Oghazi P Laven D. Exploring the dynamics of innovation: patterns of growth and contraction in the local food industry. British Food J. (2024) 126:1–7. 10.1108/BFJ-06-2023-0491

[30] EkoMatCentrum. Ekomatsligan. Ranking of Swedish municapilites based on their certified organic purchases. (2023). Available at: http://ekomatcentrum.se/ekomatsligan/ (accessed October 8, 2024).

[31] Galli A Pires SM Iha K Alves AA Lin D Mancini MS. Sustainable food transition in Portugal: Assessing the Footprint of dietary choices and gaps in national and local food policies. Sci Total Environ. (2020) 749:141307. 10.1016/j.scitotenv.2020.141307 32846345 PMC7414783

[32] Gaitán-Cremaschi D Klerkx L Aguilar-Gallegos N Duncan J Pizzolón A Dogliotti S. Public food procurement from family farming: a food system and social network perspective. Food Policy. (2022) 111:102325. 10.1016/j.foodpol.2022.102325

[33] Swensson L Hunter D Schneider S Tartanac F. Public food procurement for sustainable food systems and healthy diets-Volume 2. FAO. (2021). 10.1016/S2542-5196(21)00176-5 34390660

[34] Jones P Comfort D Hillier D. A case study of local food and its routes to market in the UK. Br Food J. (2004) 106:328–35. 10.1108/00070700410529582

[35] Enthoven L Van den Broeck G. Local food systems: Reviewing two decades of research. Agric Syst. (2021) 193:103226. 10.1016/j.agsy.2021.103226

[36] Herrero M Hugas M Lele U Wirakartakusumah A Torero M. Strengthening the sustainability of European food chains through quality and procurement policies. Trends Food Sci Technol. (2022) 120:248–53. 10.1016/j.tifs.2021.11.02138285850

[37] Smith J Andersson G Gourlay R Karner S Mikkelsen BE Sonnino R. Balancing competing policy demands: the case of sustainable public sector food procurement. J Clean Prod. (2016) 112:249–56. 10.1016/j.jclepro.2015.07.065

[38] Öhman D. (ed.). Landsbygdsminister: Köttkonsumtion inget miljöproblem. Radio Interview in Sveriges Radio (2016).

[39] Öhman D. Ministerns klimatråd om maten ifrågasätts av forskare. Radio Interview in Sveriges Radio (2023).

[40] Naturvårdsverket. Konsumtionsbaserade utsläpp av växthusgaser i Sverige och andra länder. Swedish Environmental Protection Agency (2023). Available at: https://www.naturvardsverket.se/data-ochstatistik/konsumtion/vaxthusgaser-konsumtionsbaserade-utslapp-i-sverige-ochandra-lander/ (accessed May 5, 2023).

[41] Burlingame B Dernini S. (eds.). Sustainable Diets and Biodiversity. Food and Agriculture Organization of the United Nations; Bioversity International (2010) p. 309.

