# Supplementary material for: Decreasing the carbon footprint of food through public procurement—A case study from the municipality of Härnösand
Source: Front Nutr. 2024 Nov 6;11:1330892. doi: 10.3389/fnut.2024.1330892 (PMC11576571; doi:10.3389/fnut.2024.1330892)
Supplement: Supplementary file 1 [file Data_Sheet_1.PDF]

The questions provide a structure for the study; however, the conversation is supposed to capture what the interview subject itself wants to emphasize regarding public precured food, as a producer, a purchaser, or a chef.

Questions to municipality of Härnösand

- What do your environmental and sustainability goals look like? (everyone answers, the kitchen starts, then purchaser and finally the municipality)
- Do you fulfill them? (Today you don't have any local suppliers, how do you think about that, CO_2e, organic food etc)?
- How do you relate to the emission targets that you have for the food served within the municipality. (0.5 kg CO_2e per meal).
- What actions do you need to take regarding the food served to achieve the goals?
    - Change menu?
    - Less meat?
    - Less waste?
    - Etc….
- What do you think is required to replace your suppliers with more sustainable and local ones, from the municipality, from procurement and in your purchasing organization?
- What do you think the reason is for why you do not get local suppliers to the public sector in your municipality?
- How is "local" defined in your municipality?
- Is there anything else the municipality can do to make it easier for smaller and local suppliers to participate in your procurements?
- Do you generally think that there is any inherent value in municipalities increasing the proportion of locally produced and organically produced food?

Questions to "good examples"

- You are a municipality that has succeeded in increasing the proportion of locally, sustainable, produced products in your procurements. Tell us more about that journey.
    - (Obstacles)
    - (Conditions)
    - (Adjustment etc.)
- What have been the effects in your sustainability work?
    - Is it something that you have also focused on or is it a consequence of the local collaboration?
- What does your sustainability work look like?
    - Sustainable alternatives to meat?
    - Less volume in terms of meat?
    - Sustainable meat?
    - Organic food?
    - Etc.

- What has been the single most important factor for your success in increasing the proportion of sustainable food in your procurements? Ex:
    - Creativity around LOU
    - Coordinated goods distribution.
    - Training of chef
    - Possible quantity
    - Tender for a unit
    - Farm visit
    - Etc.
- What do you think could contribute to further increasing the representation of local food (sustainable food) in your purchases? (Discussion around the same options as above).
- What has been your argument for increasing local (sustainable) food?
- What is the biggest obstacle for you to be able to control 100% of the food that crosses the threshold of your kitchen today? (Cf. a "dream scenario". Laws, finances, time, human resources, children's preferences, etc.)
- From the purchasing side, what do you think are the biggest obstacles for SMEs to deliver to the public sector? (Refer to the Swedish food strategy. Their view on the matter).
- What do you generally think is the biggest intrinsic value in municipalities increasing the proportion of locally produced food?

Questions to supplier

- Have you delivered to the public sector?
- Could you imagine being a supplier to the public sector?
- What do you want from the municipality for you to consider being a supplier to the public sector?
- What do you think the biggest obstacles for SMEs to deliver to the public sector are?
- What do you think the municipality can change in its procurement to make it easier to participate?
    - Ex….
    - Split the tender request into several geographical zones
    - Coordinated goods distribution – the production kitchen is the hub. Not packaging.
    - Negotiated procedure (turn to the market)
    - Require the possibility of study visits for school classes
    - e-procurement (do you have it today?)
    - Introduce "possible amount"
    - Submit tenders to a unit
- Have you had a dialogue with Härnösand municipality regarding an impending procurement?
- Do you generally think that there is any inherent value in municipalities increasing the proportion of locally produced and organically produced food?
